# ERBB1/2/3 Expression, Prognosis, and Immune Infiltration in Cutaneous Melanoma

**DOI:** 10.3389/fgene.2021.602160

**Published:** 2021-03-01

**Authors:** Shougang Liu, Rong Geng, Eryi Lin, Peizhen Zhao, Yongfeng Chen

**Affiliations:** ^1^Department of Dermatology, Dermatology Hospital, Southern Medical University, Guangzhou, China; ^2^Department of Gynecology and Obstetrics, The First Affiliated Hospital, Jinan University, Guangzhou, China; ^3^Department of Gynecology, Affiliated Foshan Maternity and Child Healthcare Hospital, Southern Medical University, Foshan, China

**Keywords:** ERBB family, immunochemistry, immune infiltrates, prognostic, cutaneous melanoma

## Abstract

**Background:**

The four ERBB tyrosine kinase family members [ERBB1 (epidermal growth factor receptor, EGFR), ERBB2 (HER2), ERBB3 (HER3), and ERBB4 (HER4)] (ERBB receptor family) have been shown, according to previous studies, to be related to the cutaneous melanoma. ERBB3 is the only member of the ERBBs that lacks tyrosine kinase activity and thus needs to dimer with other tyrosine kinases receptors to trigger the signaling pathway, while ERBB3 may dimer with all members of the ERBB family. Melanoma progression depends on activation of ERBB signaling, especially the ERBB3/ERBB2 cascade. There are lymphocytes and T cell infiltrates in melanoma. Numerous pieces of evidences indicate that local immune status plays an important role in the formation of anti-tumor immune responses. However, the relationship between the ERBBs and prognosis and immune infiltration in cutaneous melanoma is not completely clear.

**Methods:**

The expression of the ERBBs was analyzed through the Oncomine database, Gene Expression Profiling Interactive Analysis (GEPIA), respectively. Immunohistochemistry of ERBBs was obtained from the Human Protein Atlas is increased before HPA database. ERBBs genes expression and mutation analysis in cutaneous melanoma from the cBioPortal. Functional annotation and Kyoto Encyclopedia of Genes and Genomes is increased before KEGG pathway enrichment analysis from the Metascape. Correlations between ERBBs and 31 genes that were close to each other and frequently altered were explored by GEPIA. Using the GEPIA database, we also investigated the relationship between ERBBs and myeloid-derived suppressor cells (MDSC) in cutaneous melanoma. The disease-free survival and different tumor stages of ERBBs were evaluated by GEPIA. The correlation of ERBBs and tumor-infiltrating immune cells and prognostic(5 years survival rates) was tested by the Tumor Immune Estimation Resource (TIMER).

**Results:**

In general, the expression levels of ERBB1/2 in cutaneous melanoma were lower than those in normal skin tissue. By contrast, the ERBB3 expression level was higher in cutaneous melanoma than in normal skin tissue. Low expression of ERBB1/2 and high expression of ERBB3 were detrimental to the 5 years survival of cutaneous melanoma patients (ERBB1: log-rank P: 0.03; ERBB2: log-rank P: 0.008; ERBB3: log-rank P: 0.039). ERBB4 expression may not affect the prognosis of patients with cutaneous melanoma. ERBBs may not play a role in the tumor stage and disease-free survival in cutaneous melanoma patients. The relationship between the ERBB family and 31 genes that were close to each other and frequently altered is demonstrated as the genes regulated by the ERBB family being mainly concentrated in the RAS/RAF/MEK/ERK signaling pathway. ERBB2 can induce infiltration of CD8^+^ T cells and B cells, while ERBB3 can induce infiltration of CD4^+^ T cells, CD8^+^ T cells, and Neutrophil cells. ERBBs are more significantly associated with M1 macrophages, dendritic cells, Th1, Th2, Th17, and Treg cellular immune markers (Cor > 0.2). ERBB2/3 were related to MDSC in cutaneous melanoma, including human mononuclear myeloid-derived suppressor cells (M-MDSC) and polymorphonuclear myeloid-derived suppressor cells (PMN-MDSC), and may influence the progression of cutaneous melanoma through MDSC, but the conclusion needs further probing.

**Conclusion:**

This study investigated the prognosis and immune infiltration of the ERBB family in cutaneous melanoma. Our results suggest that ERBB1/2/3 may serve as early prognostic markers and potential therapeutic targets in cutaneous melanoma.

## Introduction

Melanoma originates from melanocytes and accounts for the highest proportion of skin cancer-related deaths. At present, the treatment of melanoma metastasis is still difficult, although researchers have explored a variety of methods. The 5 years overall survival (OS) of patients with early melanoma is higher, ranging from 24 to 29%, but the 5-year OS of patients with stage IIIC and IV is only 10–19%. In recent years, biological immunotherapy has gradually emerged, which can prolong the survival period of patients (Homet Moreno and Ribas, 2015). Therefore, further searching for potential pathogenic factors and pathogenic mechanisms can help identify potential prognostic markers and drug targets in melanoma.

The ERBB family belongs to the tyrosine kinase I subfamily and is composed of four closely related transmembrane tyrosine kinase receptors, all of which are encoded by the proto-oncogene HER1–4: ERBB1 (EGFR), ERBB2 (HER2), ERBB3 (HER3), and ERBB4 (HER4). They all have tyrosine kinase activity and play a key role in signal transduction. The tyrosine kinase active domain of each member of the ERBBs family is highly conserved, with a high degree of homology in structure and function. It is mainly expressed during human embryonic development and regulates the growth, survival, transformation, and apoptosis of normal (Jørgensen and Hersom, 2012; Griffin and Ramirez, 2017).

The overexpression and activation of the ERBB family are closely related to the clinicopathological characteristics and prognosis of various cancers, including melanoma, lung cancer, gastric cancer, breast cancer, etc. (Bittoni et al., 2015; Oudard et al., 2015). It is generally believed that abnormally functional carcinogenic pathways lead to melanoma. These pathways may include the EMT signaling pathway (Pietraszek-Gremplewicz et al., 2019), PI3K signaling pathway (Mujoo et al., 2014; Song et al., 2015), and so on. ERBBs can enhance the ability of tumor cells to migrate and invade, promote tumor angiogenesis, and inhibit tumor cell apoptosis (Iqbal and Iqbal, 2014) through these pathways.

Previous studies have shown that ERBBs disorders are closely related to the clinicopathological characteristics and prognosis of human cutaneous melanoma. However, the potential role of ERBBs family members in cutaneous melanoma remains unintelligible. In this study, we analyzed the expression, mutation, prognosis, and immune infiltration of ERBBs family members in cutaneous melanoma through a variety of databases.

## Materials and Methods

### Oncomine Database Analysis

Oncomine^1^ is an online bioinformatics analysis tool that includes 18,000 cancer gene expression microarrays (Rhodes et al., 2007). Through the Gene Summary view in the Oncomine database, we determined the expression level of ERBB in cutaneous melanoma. Use the following values: *P*-value 0.01, fold change 2, top 10% of the gene ranking, and mRNA data type to determine the threshold.

### GEPIA Database Analysis

Online database GEPIA^2^ is an online database utilized to analyze the expression data of RNA sequencing in TCGA and GTEx projects. It can also generate gene Expression profiles, the expression in the box plot, and the main stages of pathology (Tang et al., 2017). The expression of ERBB was determined by the SKCM data set of GEPIA. The following values were used to determine the threshold: the *P*-value was 0.01, the multiple changes were 2, and it matched the normal value of TCGA and GTEx data.

### Human Protein Atlas Database Analysis

The HPA (version 19.3)^3^ (Navani, 2016) is a large-scale research project, the database will help researchers to explore protein expression in human tissue and cells. In this study, immunohistochemical images were used to analyze ERBBs protein expression in cutaneous melanoma and normal tissues.

### cBioPortal Database Analysis

cBioPortal^4^ was used to further analyze the expression of ERBB (Gao et al., 2013) through the skin melanoma data set, which included 479 pathological reports. Co-expression and network analysis were performed based on the online instructions of cBioPortal.

### Functional Annotation and KEGG Pathway Enrichment Analysis

Gene function annotation and KEGG pathway analysis were used to uncover the underlying mechanism of cutaneous melanoma. MetaScape^5^ (Zhou et al., 2019) was updated in 2018 and is a web-based tool that provides gene function annotation and enrichment analysis.

### TIMER Database Analysis

The TIMER^6^ is used to study the expression characteristics of tumor-immune interaction genes in more than 30 cancer types (Li et al., 2017) to evaluate various the clinical impact of different immune cells of immune type. We analyzed the relationship between the expression of ERBBs and immune infiltration in cutaneous melanoma through the TIMER database.

### Statistical Analysis

The results we generate using Oncomine were displayed by *P*-values, fold changes, and ranks (*p* < 0.05, fold change > 2). P-value and fold change were used to show the outcomes of GEPIA (*p* < 0.01, fold change > 2). Also, the Spearman correlation analysis is used to evaluate the relationship between genes and determine the strength of the correlation between genes by absolute value. *P* < 0.05 were considered statistically significant.

## Results

### Transcriptional Levels of ERBBs in Cutaneous Melanoma and Other Cancers

**FIGURE 1:**
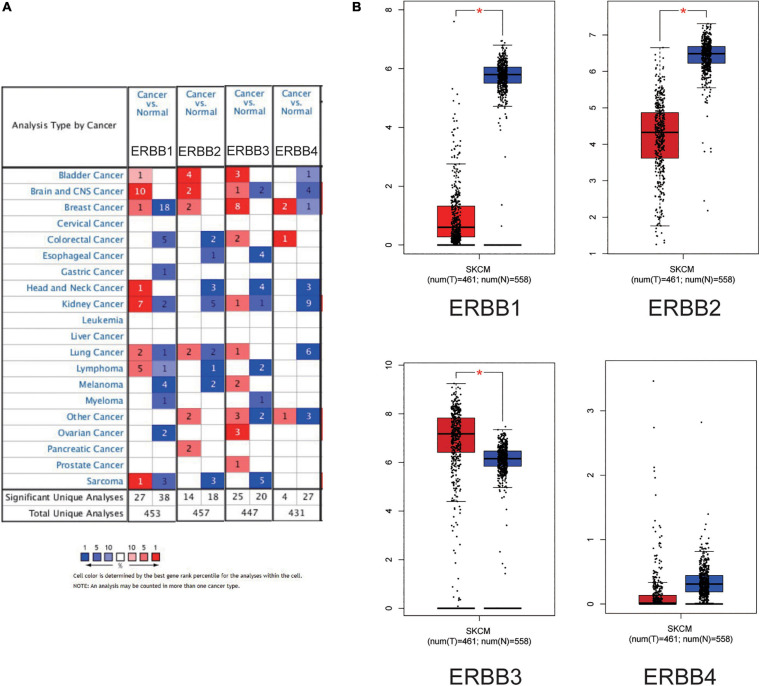
Expression of the ERBB receptor family in different types of human cancers. **(A)** High or low expression of ERBB receptor family in different human cancer tissues compared with normal tissues (ONCOMINE). The mRNA expression levels of ERBB1 and ERBB2 were decreased, but the mRNA expression level of ERBB3 was increased in cutaneous melanoma. **(B)** The expression of ERBBs in cutaneous melanoma (GEPIA) is consistent with ONCOMINE.

**FIGURE 2:**
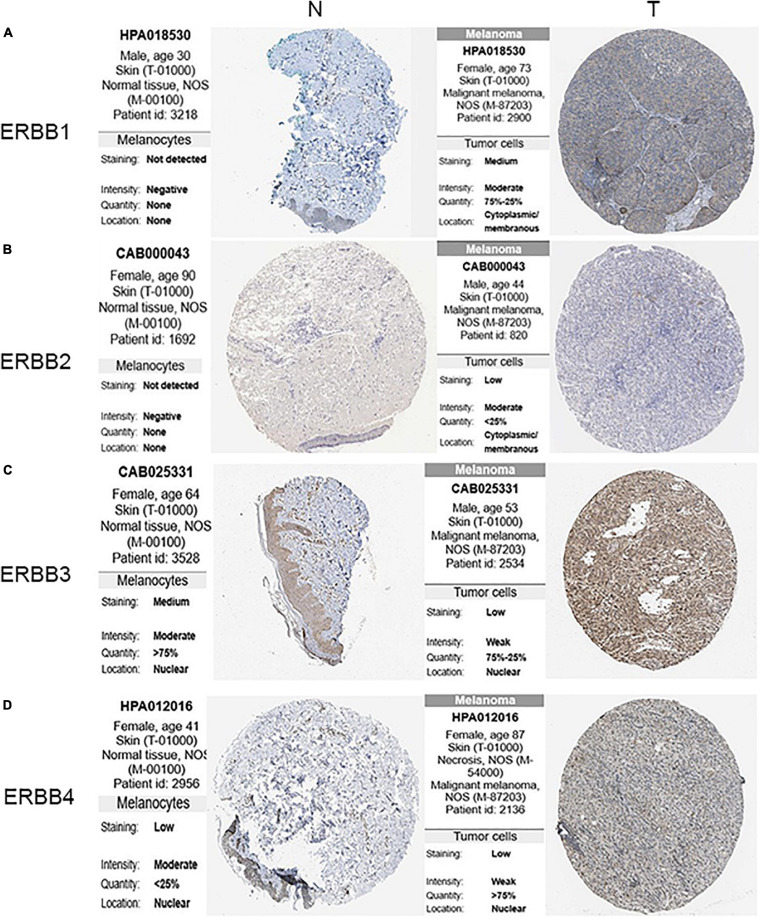
Expression of ERBB receptor family in the cutaneous melanoma (HPA). **(A)** The expression of ERBB1 protein was moderate in the cutaneous melanoma tissues, and **(B)** ERBB2 was lower in the cutaneous melanoma tissues. **(C,D)** The expression of ERBB3/4 proteins were higher in the cutaneous melanoma tissues than that in the normal skin tissues by the HPA database (N: normal; T: tumor).

The transcription levels of ERBBs were compared in cutaneous melanoma and normal samples by the ONCOMINE database (Figure 1A and Table 1). ERBB3 was upregulated in cutaneous melanoma in two datasets. In Riker’s and Talantov’s datasets (Talantov et al., 2005; Riker et al., 2008), ERBB3 is overexpressed compared with the normal samples: cutaneous melanoma with a fold change of 4.667 and 2.264, respectively. In Riker’s dataset (Riker et al., 2008), ERBB1 (EGFR) is under-expressed in cutaneous melanoma with a fold change of −8.110. Talantov’s dataset (Talantov et al., 2005) revealed another mRNA expression ERBB1 with a fold change of −7.657; that is, ERBB2 has a fold change of −3.952 and −2.229 in cutaneous melanoma compared with normal skin tissues in Riker’s and Talantov’s datasets, respectively. Based on the information afforded by the GEPIA database, a comparative study of ERBBs mRNA expression in normal skin tissue and cutaneous melanoma tissue was carried out. The results showed that, compared with normal skin tissue, cutaneous melanoma tissue had lower expression levels of ERBB1/2 and a higher expression level of ERBB3 (Figure 1B). In the immunohistochemistry supplied by the HPA data set, we discovered that ERBB1 was moderately expressed, ERBB2 was lowly expressed, and the ERBB3/4 proteins were highly expressed in cutaneous melanoma (Figure 2).

**TABLE 1:** Conspicuous variation of ERBBs expression in transcription level between cutaneous melanoma and skin tissues (ONCOMINE database).

	Type vs. normal	Fold change	*P*-value	*t*-test	Reference
ERBB1	Cutaneous melanoma vs. normal	−8.110	2.44E-8	−10.259	Riker
	Cutaneous melanoma vs. normal	−7.657	1.84E-12	−16.418	Talantov
ERBB2	Cutaneous melanoma vs. normal	−3.952	2.70E-6	−6.762	Riker
	Cutaneous melanoma vs. normal	−2.229	5.86E-5	−6.116	Talantov
ERBB3	Cutaneous melanoma vs. normal	4.667	4.73E-9	12.658	Talantov
	Cutaneous melanoma vs. normal	2.264	0.006	2.891	Riker
ERBB4	NA	NA	NA	NA	NA

*Fold change >> 2, P-value< < 0.05.*

Further,we explored the expression of the ERBBs in other cancers models by ONCOMINE database (Figure 1A) showed that ERBB1 mRNA level was significantly higher in the bladder, brain and CNS, head and neck, kidney, lung, lymphoma cancer tissues, and markedly lower in the breast, colorectal, gastric, myeloma, ovarian, and sarcoma cancers compared with the corresponding normal tissues. The ERBB2 mRNA level was observably higher in the bladder, brain and CNS, breast, and pancreatic cancer tissues and signally lower in the colorectal, esophageal, head and neck, kidney, lung, lymphoma, and sarcoma cancers compared with the corresponding normal tissues. The ERBB3 mRNA level was dramatically higher in bladder, colorectal, kidney, lung, ovarian, and prostate cancer tissues and significantly lower in the brain and CNS, esophageal, head and neck, lymphoma, myeloma, and sarcoma cancers compared with the corresponding normal tissues. The ERBB4 mRNA level was higher in the breast and colorectal cancer tissues and observably lower in the bladder, brain and CNS, head and neck, kidney, and lung cancers compared with the corresponding normal tissues. These results show that ERBB1/2 are of low expression, and ERBB3 is of high expression, which suggests that ERBB1/2/3 may be potential prognostic markers in cutaneous melanoma.

### ERBBs Mutation Rates, Their Influence on Neighboring Genes, and Their Correlation in Cutaneous Melanoma

**FIGURE 3:**
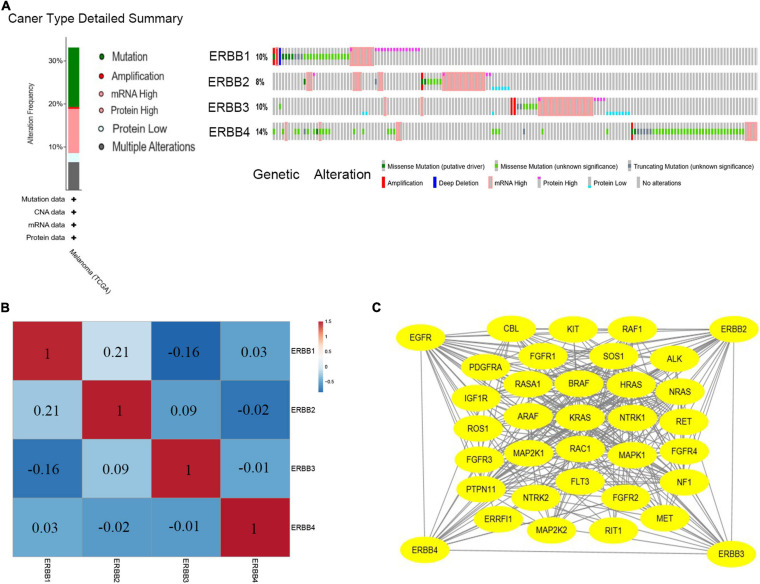
ERBBs genes expression and mutation analysis in cutaneous melanoma (cBioPortal). **(A)** ERBB1, ERBB2, ERBB3, and ERBB4 mutation rates were 10, 8, 10, and 14%, respectively. **(B)** Calculate the pertinence of the ERBB receptor family with each other by analyzing their mRNA expression, positive correlations were detected in ERBB1and ERBB2. **(C)** Network for ERBB receptor family and the 31 most frequently altered neighbor genes.

**FIGURE 4:**
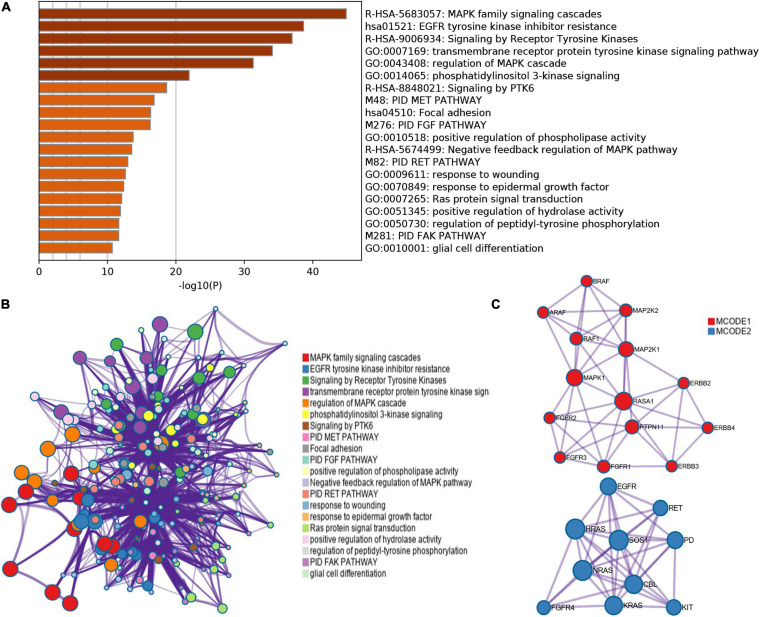
GO and KEGG analysis of ERBB receptor family (Metascape). **(A)** Bar graph of enriched terms across these enriched genes in patients with cutaneous melanoma, colored by *p*-values. **(B,C)** A network of enriched terms: **(B)** colored by cluster-ID, where nodes that share the same cluster-ID are typically close to each other; **(C)** Protein–protein interaction network and MCODE components identified in the genes enriched in patients with cutaneous melanoma.

cBioPortal is used to analyze the changes and network of ERBBs in cutaneous melanoma. In 479 cases, ERBBs were changed in 158 samples (33%), and two or more changes were detected in 53 samples (11%) (Figure 3A). Besides, we analyzed the mRNA expression of ERBBs (RNA Seq V2 RSEM) through Pearson correlation to calculate the relationship between ERBBs, and the results showed that ERBB1 and ERBB2 are positively correlated (Figure 3B). Subsequently, we established a network of ERBBs through the 31 most frequently changing neighbor genes [The Network TAB provides interaction analysis and network visualization of cancer changes in cBioPortal, the network includes Pathway, HPRD (human reference protein database), Reactome, NCI (National Cancer Institute)—Nature Pathway Interaction Database, Memorial Sloan Kettering Cancer Center (MSKCC), map of cancer cells^7^, From The Open Source Pathway Commons Project. By default, cBioportal automatically generates a network containing all queries about the genetic neighbors (adjacent nodes), sequenced according to the alternating frequency of the genome of the selected cancer]. This shows that there is a close pertinence between changes of ERBBs and cell proliferation or differentiation, Fibroblast growth factor receptor (FGFR), including FGFR1/2/3/4, as well as MAPK pathways-related genes, including MAPK1, MAP2K1, and MAP2K2 (Figure 3C). Evaluate the function of clustering genes through GO and pathway analysis, and these enriched pathways are closely interrelated to each other. Figure 4 provides the consequence of the functions and pathways of the markedly enriched genes. We discovered that the enriched genes are related to multiple pathways, such as MAPK family signal cascade, MAPK cascade regulation, EGFR tyrosine kinase inhibitor resistance, receptor tyrosine kinase signal transduction, and transmembrane receptor protein tyrosine kinase signal transduction pathway, etc.

Further, through the GEPIA database (Supplementary Table 1), in cutaneous melanoma, we examined the association between the ERBBs and 31 genes were close to each other and frequently altered and observed that all the genes except KIT, ALK, NTRK1, FGFR4, and MET were affected by the changes of some ERBB family members. In this network, ERBB-regulated genes are mainly concentrated in the RAS-RAF-MEK-ERK signaling pathway, while the ERBB family may have no direct regulatory relationship with SOS, NF1, PDGFRA, FGFR1, RASA1, ROS1, FGFR3, RAC1, PTPN11, NTRK2, FLT3, FGFR2, ERRFI1, and RIT1 genes.

### Immune Cell Infiltration of ERBBs in Cutaneous Melanoma

**FIGURE 5:**
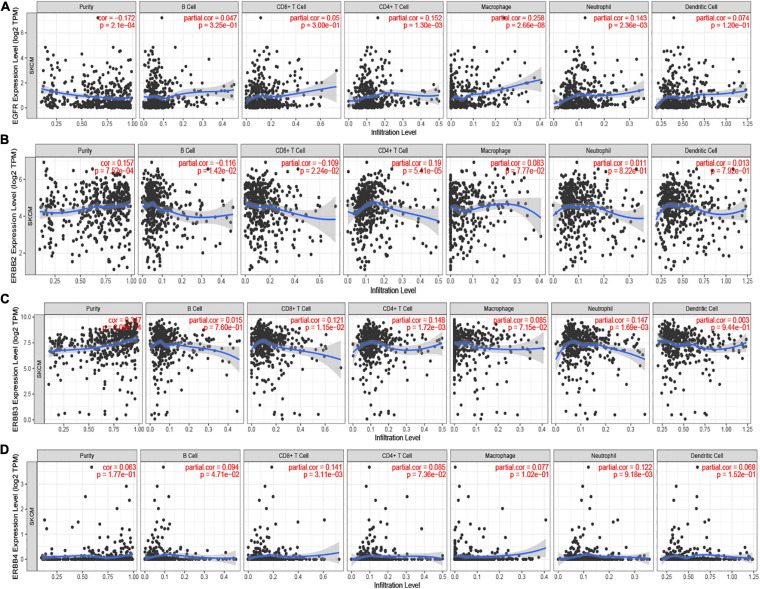
The Cox proportional hazard model of ERBBs and six tumor-infiltrating immune cells in cutaneous melanoma (TIMER). **(A)** ERBB1 (EGFR) expression had noteworthy positive interrelated to infiltrating levels of CD4^+^ T cells, Macrophage, and Neutrophil. **(B)** ERBB2 was negatively correlative with the infiltration of B cells and CD8^+^ T cells but positively related to the infiltration of CD4^+^ T cells. **(C)** ERBB3 expression was positively associated with the infiltration of CD8^+^ T cells, CD4^+^ T cells, and Neutrophil. **(D)** ERBB4 expression was positively related to the infiltration of CD8^+^ T cells and neutrophils. The correlation between ERBBs and immune cells were used to analyze by Spearman’s correlation.

ERBBs affect the clinical prognosis of patients by participating in the inflammatory response and immune cell infiltration. TIMER displays the relationship between ERBBs and immune infiltrating cells. There was a positive pertinence between ERBB1 expression and the infiltration of CD4^+^ T cells (Cor = 0.152, *p* = 1.30e-03), Macrophages (Cor = 0.258, *p* = 2.66e-08), and Neutrophils (Cor = 0.143, *p* = 2.36e-03; Figure 5A). ERBB2 expression was negatively related to the infiltration of B cells (Cor = −0.116, *p* = 1.42e-02) and CD8^+^ T cells (Cor = −0.109, *p* = 2.24e-02) and positively associated with the infiltration of CD4^+^ T cells (Cor = 0.19, *p* = 5.39e-05; Figure 5B). ERBB3 expression was positively interrelated to the infiltration of CD8^+^ T cells (Cor = 0.121, *p* = 1.15e-02) and CD4^+^ T cells (Cor = 0.148, *p* = 1.72e-03) and Neutrophil (Cor = 0.147, *p* = 1.69e-03; Figure 5C). There was a positive correlation between ERBB4 expression and the infiltration of CD8^+^ T cells (Cor = 0.141, *p* = 3.12e-03) and neutrophils (Cor = 0.122, *p* = 9.21e-03; Figure 5D). These results reveal that ERBB2/3 are more closely related to immune infiltration in cutaneous melanoma, which suggests that the role of ERBB2/3 in regulating tumor immunity.

### ERBBs CNV Is Correlated With Immune Infiltration Levels in Cutaneous Melanoma

**FIGURE 6:**
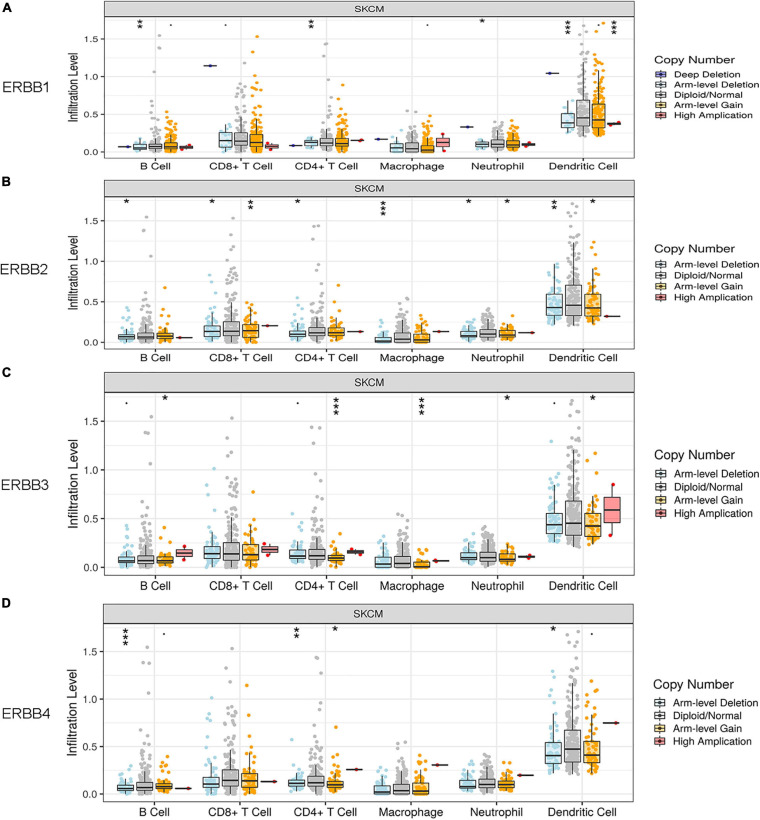
Relationship between ERBBs CNV and invasion levels of B cells, CD8^+^ T cells, CD4^+^ T cells, macrophages, neutrophils, and dendritic cells in cutaneous melanoma. **(A)** ERBB 1(EGFR) affects the infiltrating levels of B cells, CD4^+^ T cells, Neutrophils, and Dendritic Cells in cutaneous melanoma. **(B)** ERBB2 affects the infiltrating levels of B cells, CD8^+^ T cells, CD4^+^ T cells, Macrophages, Neutrophils, and Dendritic Cells. **(C)** ERBB3 affects the infiltrating levels of B cells, CD4^+^ T cells, Macrophages, Neutrophils, and Dendritic Cells. **(D)** ERBB4 affects the infiltrating levels of B cells, CD4^+^ T cells, and Dendritic Cells.

ERBB CNV has a signal related to infiltrating levels of B cells, CD8^+^ T cells, CD4^+^ T cells, Macrophages, Neutrophils, and Dendritic Cells. ERBB 1 (EGFR) induces the infiltrating levels of B cells, CD4^+^ T cells, Neutrophils, and Dendritic Cells in cutaneous melanoma (Figure 6A). ERBB2 induces the infiltrating levels of B cells, CD8^+^ T cells, CD4^+^ T cells, Macrophages, Neutrophils, and Dendritic Cells (Figure 6B). ERBB3 induces the infiltrating levels of B cells, CD4^+^ T cells, Macrophages, Neutrophils, and Dendritic Cells (Figure 6C). ERBB4 induces the infiltrating levels of B cells, CD4^+^ T cells, and Dendritic Cells (Figure 6D). ERBB1/2/4 CNV are correlated with arm-level deletion. ERBB3 CNV is connected with arm-level gain. These results suggest that ERBBs CNV induces immune infiltration in cutaneous melanoma.

### Correlation Analysis Between ERBBs and Markers of Immune Cells in Cutaneous Melanoma

We further evaluated the relationship between the ERBBs levels and immune infiltrating cells through the TIMER database and according to the expression levels of immune marker genes in cutaneous melanoma tissues. The immune cells analyzed by the TIMER database include CD8^+^ T cells, CD4^+^ T cells, B cells, monocytes, tumor-associated macrophages (TAM), M1 and M2 macrophages, neutrophils, dendritic cells, and different subgroups of T cells, namely, T helper 1 (Th1), Th2, Tfh, Th17, regulatory T (Tregs), and T cell exhaustion. Because tumor purity will affect the level of immune infiltration of clinical samples, the purity of the relevant analysis was adjusted (Table 2).

**TABLE 2:** Correlation analysis between ERBBs and related markers of immune cells.

Description	Gene markers	SKCM							
		Purity							
		ERBB1		ERBB2		ERBB3		ERBB4	
		Cor	*P*	Cor	*P*	Cor	*P*	Cor	*P*
CD8^+^ T cell	CD8A	−0.053	2.62e-1	−0.045	3.37e-01	0.133	**	0.1	*
	CD8B	−0.078	9.74e-02	−0.066	1.61e-01	0.086	6.62e-02	0.086	6.65e-02
T cell (general)	CD3D	−0.041	3.85e-01	−0.088	5.90e-02	0.047	3.18e-01	0.08	8.59e-02
	CD3E	−0.029	5.32e-01	−0.05	2.85e-01	0.047	3.11e-01	0.087	6.26e-02
	CD2	−0.015	7.48e-01	−0.066	1.58e-01	0.102	*	0.107	*
B cell	CD19	−0.007	8.85e-01	−0.117	*	0.021	6.57e-01	0.155	***
	CD79A	−0.094	*	−0.111	*	0	9.98e-01	0.13	**
Monocyte	CD86	0.072	1.22e-01	−0.001	9.85e-01	0.062	1.85e-01	0.151	**
	CD115	0.175	***	0.102	*	0.08	8.57e-02	0.123	**
TAM	CCL2	0.128	**	0.009	8.52e-01	−0.021	6.57e-01	0.11	*
	CD68	−0.015	7.50e-01	−0.041	3.77e-01	−0.147	**	−0.054	2.49e-01
	IL10	0.063	1.77e-01	−0.016	7.27e-01	0.002	9.63e-01	0.056	2.31e-01
M1 Macrophage	iNOS	−0.205	***	0.012	8.01e-01	0.044	3.43e-01	−0.006	8.92e-01
	IRF5	0.073	1.21e-01	0.033	4.86e-01	−0.076	1.07e-01	−0.006	8.94e-01
	COX2	0.215	***	0.043	3.57e-01	−0.006	8.94e-01	0.127	**
M2 Macrophage	CD163	0.124	**	0.045	3.40e-01	0.047	3.17e-01	0.148	**
	VSIG4	0.063	1.77e-01	0.073	1.20e-01	0.05	2.84e-01	0.106	*
	MS4A4A	0.091	5.30e-02	−0.007	8.74e-01	0.044	3.43e-01	0.139	**
Neutrophils	CD66b	0.049	2.94e-01	−0.062	1.88e-01	−0.116	*	0	9.94e-01
	CD11b	0.104	*	0.062	1.84e-01	0.085	7.00e-02	0.102	*
	CCR7	0.042	3.73e-01	−0.076	1.07e-01	0.06	2.01e-01	0.1	*
Dendritic cell	HLA-DPB1	0.036	4.42e-01	0.113	*	0.107	*	0.052	2.65e-01
	HLA-DQB1	0.05	2.91e-01	0.039	4.11e-01	0.066	1.58e-01	0.068	1.46e-01
	HLA-DRA	0.046	3.26e-01	0.055	2.42e-01	0.115	*	0.084	7.19e-02
	HLA-DPA1	0.027	5.67e-01	0.1	*	0.119	*	0.064	1.69e-01
	BDCA-1	0.214	***	−0.01	8.26e-01	0.084	7.32e-02	0.162	***
	BDCA-4	0.434	***	0.079	9.06e-02	0.053	2.59e-01	0.182	***
	CD11c	0.011	8.17e-01	−0.029	5.41e-01	−0.059	208e-01	0.067	1.54e-01
Th1	T-bet	−0.017	7.22e-01	−0.06	2.02e-01	0.066	1.56e-01	0.121	**
	STAT4	−0.114	*	0.007	8.83e-01	0.181	***	0.172	***
	STAT1	−0.01	8.25e-01	0.043	3.56e-01	0.242	***	0.107	*
	IFN-γ	−0.068	1.45e-01	−0.102	*	0.107	*	0.084	7.22e-02
	TNF-α	0.106	*	0.026	5.73e-01	0.046	3.25e-01	0.026	5.85e-01
Th2	GATA3	0.327	***	0.089	5.76e-02	0.015	7.42e-01	0.144	**
	STAT6	0.141	**	0.228	8.32e-07	0.337	***	0.069	1.40e-01
	STAT5A	−0.045	3.33e-01	0.159	***	0.326	***	0.103	**
	IL13	0.049	2.93e-01	−0.052	2.68e-01	−0.018	7.06e-01	0.007	8.80e-01
Tfh	BCL6	0.255	***	0.158	***	0.023	6.27e-01	0.158	***
	IL21	0.08	8.66e-02	0.077	1.01e-01	0.057	2.27e-01	0.179	***
Th17	STAT3	0.282	***	0.331	***	0.29	***	0.195	***
	IL17A	0.034	4.63e-01	0.079	9.02e-02	0.003	9.50e-01	−0.037	4.28e-01
Treg	FOXP3	0.072	1.22e-01	−0.013	7.76e-01	−0.004	9.28e-01	0.044	3.44e-01
	CCR8	0.138	**	−0.039	4.09e-01	0.145	**	0.133	**
	STAT5B	0.174	***	0.235	***	0.331	***	0.174	***
	TGFβ	0.276	***	0.177	***	−0.1	*	0.048	3.02e-01
T cell exhaustion	PD-1	−0.099	*	−0.048	3.01e-01	0.065	1.66e-01	0.063	1.78e-01
	CTLA4	−0.086	6.49e-02	−0.171	***	−0.076	1.05e-01	0.071	1.29e-01
	LAG3	−0.129	**	−0.034	4.65e-01	0.058	2.19e-01	0.053	2.59e-01
	TIM-3	0.036	4.42e-01	0	9.94e-01	0.069	1.41e-01	0.118	**
	GZMB	−0.097	*	−0.132	**	−0.011	8.17e-01	0.016	7.31e-01

TAM, tumor-associated macrophage; Th, T helper cell; Tfh, Follicular helper T cell; Treg, regulatory T cell; Cor, R-value of Spearman’s correlation; Purity, correlation adjusted by purity. *P < 0.05, **P < 0.01, ***P < 0.001.

Specifically, ERBB1 expression displayed dramatically interrelated to the expression of specific immune cells markers, such as B cell marker, CD79A, Monocyte marker, CD115, TAM marker, CCL2, M1 macrophage marker, iNOS and COX2, M2 Macrophage marker, CD163, Neutrophils marker, CD11B, Dendritic cell marker, BDCA-1 and BDCA-4.Th1 markers, STAT4 and TNF-α, Th2 markers, GATA3 and STAT6, the Tfh marker, BCL6, Th17 and STAT3, Treg markers, CCR8, STAT5 and TGFβ, T cell exhaustion markers, PD-1, LAG3, and GZMB. The memorable pertinence between ERBB2 expression and the expression of specific immune cells markers is shown, such as the B-cell marker, CD19 and CD79a, Monocyte marker, CD115, Dendritic cell marker, HLA-DPB1 and HLA-DPA1, Th1 marker IFN-γ, Th2 marker, STAT5A, Tfh marker, BCL6, Th17 marker, STAT3, Treg marker, CCR8, STAT5B, and TGFβ, T cell exhaustion marker, CTLA4, and GZMB. ERBB3 expression indicated significantly related to the expression of specific immune cells markers, such as the CD8^+^ T cell marker, CD8A, T cell (general) marker, CD2, TAM marker, CD68, Neutrophils, CD66b, Dendritic cell, HLA-DPB1, HLA-DRA and HLA-DPA1, Th1 marker, STAT4, STAT1 and IFN-γ, Th2 marker, STAT6 and STAT5, Th17 marker, STAT3, Treg marker, CCR8, STAT5B, and TGFβ. ERBB4 expression manifested memorably relevant to the expression of specific immune cells markers, such as the CD8^+^ T cell marker, CD8A, T cell (general) marker, CD2, B cell marker, CD19, CD79A, Monocyte marker, CD86, CD115, TAM marker, CCL2, M1 Macrophage marker, COX2, M2 Macrophage marker, CD163, VSIG4, MS4A4A, Neutrophils marker, CD11b, and CCR7, Dendritic cell marker, BDCA-1 and BDCA-4, Th1 marker, T-bet, STAT4 and STAT1, Th2 marker, GATA3 and STAT5A, Tfh marker, BCL6 and IL21, Th17 marker, STAT3, Treg marker, CCR8 and STAT5B, T cell exhaustion marker, and TIM-3. The study shows that the ERBBs and M1 Macrophage, Dendritic cell, Th1, Th2, Th17, and Treg cells have a more significant relationship (Cor > 0.2) in cutaneous melanoma.

### Expression of ERBBs With Tumor Stage and Survival Outcome in Cutaneous Melanoma

**FIGURE 7:**
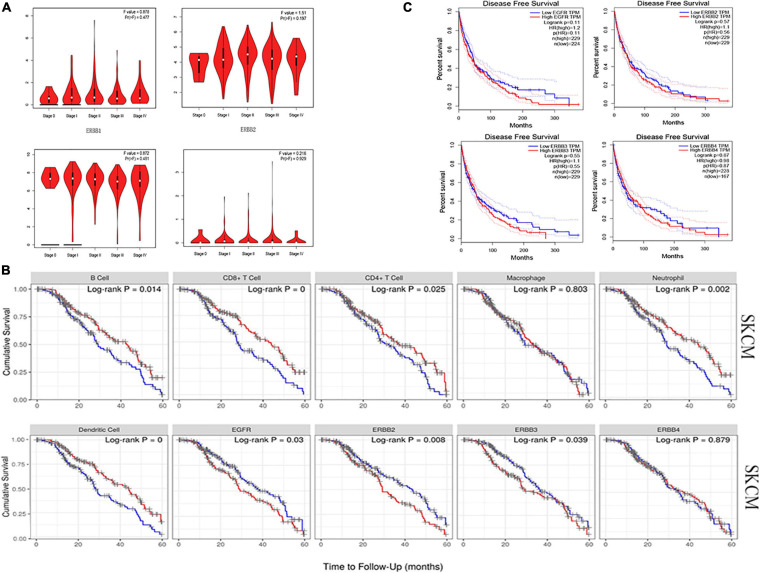
Relationship between ERBB receptor family and tumor stage and survival outcome in cutaneous melanoma. **(A)** The ERBB receptor family may not affect the tumor stage in cutaneous melanoma patients (GEPIA). **(B)** Kaplan-Meier analysis uncovers that immune cells (B cells, CD8^+^ T cells, CD4^+^ T cells, Macrophages, Neutrophils, and Dendritic Cells), ERBB1 (EGFR), ERBB2, and ERBB3 impact the 5 years survival rates of patients with cutaneous melanoma (TIMER). **(C)** ERBB receptor family may not influence disease-free survival in cutaneous melanoma.

We analyzed the relationship between ERBBs expression and tumor stage and disease-free survival, whereas ERBBs may not affect tumor stage and disease-free survival in cutaneous melanoma (Figures 7A,C). Also, we found that ERBB1/2/3 and immune infiltration cells (B cells, CD4^+^ T cells of CD8^+^ T cells, macrophages, neutrophils, and dendritic cells) influenced the 5 years survival of patients with cutaneous melanoma, while ERBB4 may not affect the 5-year survival of cutaneous melanoma patients (Figure 7B). These results show that the expression of ERBB1/2/3 in early cutaneous melanoma have prognostic significance.

### ERBB2/3 Are Associated With Myeloid-Derived Suppressor Cells (MDSC) in Cutaneous Melanoma

In the GEPIA database, we found that ERBB2 was positively correlated with Human monocytic myeloid-derived suppressor cell(s) (M-MDSC) markers CD14 (Table 3), while ERBB3 was negatively related to Human M-MDSC markers CD11b and CD14. ERBB1 was positively correlated with Human PMN-MDSC markers ARG1, CXCR2, and negatively relevant to CD63. ERBB2 was negatively connected with Human PMN-MDSC markers CD62L and CD63 and positively correlated with ARG1 and CXCR2. ERBB3 was negatively correlated with Human PMN-MDSC markers, CD62L, CD274, CXCR2, and CXCR4 and positively correlated with CD63. ERBB4 was positively associated with Human PMN-MDSC marker CD54. These results suggest that ERBB2/3 may be closely related to MDSC (Human M-MDSC and Human PMN-MDSC) in cutaneous melanoma. In terms of specific mechanisms, further research is needed in the future.

**TABLE 3:** ERBB2/3 is associated with myeloid-derived suppressor cells (MDSC) in cutaneous melanoma.

SKCM									
Description	Gene markers	ERBB1		ERBB2		ERBB3		ERBB4	
		Cor	*P*	Cor	*P*	Cor	*P*	Cor	*P*
Human M-MDSC	CD11b	0.038	0.41	0.068	0.14	−0.093	*	0.061	0.19
	CD14	0.026	0.57	0.099	*	−0.19	***	0.023	0.61
Human PMN-MDSC	CD66b	0.032	0.49	−0.032	0.49	−0.026	0.58	−0.023	0.62
	CD33	−0.0095	0.84	0.072	0.12	−0.013	0.77	0.076	0.1
	CD62L	−0.0065	0.89	−0.14	**	−0.19	***	−0.0089	0.85
	CD54	−0.077	0.1	−0.022	0.64	−0.054	0.25	0.15	**
	CD63	−0.13	**	−0.12	**	0.26	***	−0.075	0.11
	CD274	0.024	0.6	−0.035	0.46	−0.12	*	−0.0075	0.87
	ARG1	0.14	**	0.28	***	−0.019	0.68	−0.03	0.52
	CXCR2	0.21	***	0.28	***	−0.15	**	−0.051	0.28
	CXCR4	0.015	0.75	−0.083	0.076	−0.19	***	0.038	0.41

*P<0.05, **P<0.01, ***P<0.001.

## Discussion

Previous studies have shown (Alaoui-Jamali et al., 2015; Elster et al., 2015) that ERBB family members can encode a type I transmembrane protein with common structural properties and be activated by homo-or hetero-dimerization with ERBB family members. For example, when ERBB3 binds to its specific ligand NRG1, it can form a heterodimer with other ERBB partners. Ng et al. (2014) found synergistic activation of ERBB2 and ERBB3 as well as synergistic activation of ERBB3 and ERBB4 in cutaneous melanoma cell lines after NRG1 stimulation. This dimerization activates PI3K/AKT and MAPK/ERK signal transduction pathways. It has been widely recognized that ERBB family members participate in tumorigenesis and tumor therapeutic resistance by activating PI3K and MAPK signaling pathways (Elster et al., 2015). ERBB3 is often highly expressed in primary melanoma and metastatic tumors, and high levels of ERBB2 and ERBB3 are often detected in BRAF WT and mutant cells. Other studies have shown that ERBB4 detected in human melanoma cells (Gordon-Thomson et al., 2005) is mainly a truncated type (120 kD) rather than a full-length protein. So far, ERBB4 has not been widely studied in melanoma. Although the significance of ERBBs has been confirmed, it is still necessary to clarify the function of ERBBs in cutaneous melanoma.

ERBB1 expression is upregulated in many cancers, but gene expression is inconsistent in cutaneous melanoma. Some studies have stated that ERBB1 over-expression often occurs in advanced stages of melanoma. The expression of ERBB1 in cutaneous melanoma tissues was lower than that in normal tissues. However, in immunohistochemistry, ERBB1 showed moderate expression. ERBB1 autophosphorylation is the key to the PI3K/AKT and MAPK pathways. ERBB1 participates in regulating cell proliferation, apoptosis, and promoting cell invasion through these cascade reactions. We found that ERBB1 is the low expression and may not be associated with tumor stage and disease-free survival but is with 5 years survival.

ERBB2 is a HER2-receptor tyrosine kinase that can cause uncontrolled cell proliferation and tumorigenesis through various mechanisms (Hynes and Lane, 2005). ERBB2 is a vital cancer marker, and no ligand for ERBB2 has been found yet. ERBB2 forms a heterodimer with another ERBB family member to form a more stable and strong signaling function and is considered to be an important therapeutic target for cancer (Giroux, 2013; Milik et al., 2017; Khanjani et al., 2018). ERBB2 is associated with poor clinical prognosis, even though its expression is low (Gilcrease et al., 2009). In our study, it was found that ERBB2 is the low expression in cutaneous melanoma tissues, but this expression may not affect tumor stage and disease-free survival; however, it is related to the 5 years survival rates of cutaneous melanoma patients.

ERBB3 plays a meaningful role in cell proliferation and survival (Yarden and Pines, 2012). Because ERBB3 lacks tyrosine kinase activity, which cannot initiate a signal cascade through autophosphorylation, it must heterodimerize with ERBB1 or ERBB2 to phosphorylate tyrosine in the C-terminal domain of ERBB3 (Campbell et al., 2010). Ueno et al. (2008) found that ERBB3 and ERBB1 dimerized in melanoma cells and promoted the metastasis of melanoma to a certain extent. ERBB3 is overexpressed and activated in a variety of cancers. The regulation of ERBB3 expression and signaling involves many HER3 interacting proteins, including PI3K, Shc, and E3 ubiquitin ligases NEDD4 and Nrdp1 (Mujoo et al., 2014). In our report, we confirmed that ERBB3 exhibits high expression and influences the 5 years survival rates of cutaneous melanoma patients but may not play role in the tumor stage and disease-free survival.

ERBB4 has been shown to have a crucial role in the normal growth of the central nervous system, breast, and fetus (Chuu et al., 2008; Liu et al., 2010; Iwakura and Nawa, 2013). However, the role of ERBB4 in human cancer is still debatable. ERBB4 tyrosine kinase activates through ligand-bound dimerization and induces activation of mitogen-activated protein kinases (MAPKs) and phosphatidylinositol kinase (PI3K)/AKT pathway (Prickett et al., 2009). Prickett et al. (2009) reported that somatic mutations of ERBB4 in malignant melanoma are widespread and proved that ERBB4 mutations are new drug targets for the treatment of metastatic melanoma. However, Zhou et al. (2013) and others reported that ERBB4 hotspot mutations were not detected in melanoma patients in southern China, suggesting that in Chinese melanoma patients, ERBB4 mutations can only play a limited role. These conclusions prove that there may be geographical differences in mutations of susceptibility genes in melanoma (Casula et al., 2009). In this chapter, we found that ERBB4 is the highest mutation rate in the ERBB family, but it may not impact the tumor stage, disease-free survival, and 5 years survival rates of melanoma patients.

We analyzed the relationship between the ERBB family and 31 genes that are close and frequently altered. In melanoma, KIT, ALK, NTRK1, FGFR4, and MET may not be regulated by the ERBB family, while the genes regulated by the ERBB family are mainly concentrated in the RAS-RAF-MEK-ERK signaling pathway (Wee and Wang, 2017), which is one of the core pathways in the pathogenesis of melanoma (McCubrey et al., 2007) and also a pharmacological target for cancer treatment. CBL, as a ubiquitin ligase, can ubiquitinate ERBB1 to activate downstream signaling pathways (Levkowitz et al., 1998; Grøvdal et al., 2004). The heterodimer combination of ERBB2 and ERBB1 or ERBB3 has strong signal activity (Pinkas-Kramarski et al., 1996), which activates downstream Ras/Raf/MEK/ERK cascade reaction pathway under the stimulation of external factors (Djerf Severinsson et al., 2011). Among them, RAS family genes include HRAS, NRAS, and KRAS; RAF family genes include ARAF, BRAF, and RAF1. ERK signaling pathway includes MAPK1, MAPK2P1, and MAPK2P2. Besides, ERBB1/IGF-1R/CRAF can reduce the proliferation of melanoma cells by inhibiting MAPK and/or PI3K/AKT signaling pathways (Sun et al., 2016). In cutaneous melanoma, the direct association of the ERBB family with other genes (SOS, NF1, PDGFRA, FGFR1, RASA1, ROS1, FGFR3, RAC1, PTPN11, NTRK2, FLT3, FGFR2, ERRFI1, and RIT1) had not been reported, and so the relationship between ERBB family and these genes remains to be further studied and determined.

In some studies, the presence of tumor-infiltrating lymphocytes (TIL) in melanoma is associated with better prognosis and has been interpreted as an indicator that the host promotes a more effective immune response to the tumor (Clemente et al., 1996; Anichini et al., 2012). However, the significance of TIL remains to be further demonstrated. Gooden et al. (2011) conducted a meta-analysis on the impact of TIL on cancer prognosis in 2011 and found that the presence of CD3^+^ and CD8^+^ cells had a beneficial impact on the survival of patients. In our study, ERBB2 can induce infiltration of CD8^+^ T cells and B cells, while ERBB3 can induce infiltration of CD4^+^ T cells, neutrophils cells, and CD8^+^ T cells. CD8^+^T cells combine antigens and MHC I molecules to form complexes. Once CD8^+^T cells are fully activated, they can induce the apoptosis of melanoma cells by releasing perforin and granules (Giavina-Bianchi et al., 2017; Zhang et al., 2017). Forsthuber et al. (2019) found that neutrophils play an environmentally dependent role in melanoma in a mouse tumor transplantation model and can be actively switched to an anti-tumor model. CD4^+^ T cells with MHC class II molecules present antigens; the combination of cytokines in the microenvironment under the action of cells can differentiate into various kinds of effects and can be turned into CD4^+^ T helper cells (Th) to activate CD8^+^ T cells, B cells, and natural killer cells (NK) to tumor cells to play an antitumor immune response (Giavina-Bianchi et al., 2017).

We investigated the relationship between ERBB and immune-infiltrating cell markers (Cor > 0.2). The downstream of EGFR, the MAPK pathway, stimulates the activation of NF-KappaB heteromorphs and homodimers to drive the expression of iNOS, thus supporting the occurrence of melanoma. COX2, as the downstream of the NF-kappa B pathway, plays a pro-oncogenic role in the NF-kB-iNOS-COX-2 signaling pathway (Uffort et al., 2009). Neuropilin-1 (NRP1, BDCA-4) induces a c-Jun N-terminal kinase (JNK)-dependent signaling cascade that leads to the upregulation of EGFR or IGF1R, thereby promoting cancer cell growth (Rizzolio et al., 2020). Studies have shown that the TGF-β pathway can initiate EGFR expression (Sun et al., 2014), making EGFR fully pathogenic. Epidermal growth factor binds to receptors and triggers a variety of signal transduction pathways, one of which activates signal transduction and transcriptional activator (STAT) (Olayioye et al., 1999). From the table we can see that ERBBs are closely related to STAT1, STAT3, STAT5A, STAT5B, and STAT6 (Cor > 0.2). ERBB1 induced low expression of STAT3, STAT5B, and STAT6. ERBB2 induced low expression of STAT3, STAT5A, and STAT5B. ERBB3 induced high expression of STAT1, STAT3, STAT5A, and STAT6. ERBB1/2 can induce low expression of STAT3 and STAT5B may be related to the formation of heterodimer combination. In malignant melanoma, Insulin-like growth factor binding protein 2 (IGFBP2) regulates the expression of PD-L1 by activating the EGFR-STAT3 signaling pathway (Li et al., 2020). The irreversible inhibition of Canertinib on ERBB1-3 was more effective in inhibiting Akt, ERK1/2, and STAT3 signaling pathways (Djerf Severinsson et al., 2011). Phosphorylated STAT5 is regulated by rEGF in melanoma, and inhibition of STAT5B expression can significantly reduce the expression of BCL-2, resulting in decreased cell survival rate and increased apoptosis (Mirmohammadsadegh et al., 2006). Studies have shown that high STAT1, STAT3, and STAT5B expression and low STAT6 expression are associated with better prognosis in SKCM patients. These studies suggest that ERBBs may be closely related to the STAT signaling pathway in cutaneous melanoma. Also, the direct relationship between ERBBs and immune-infiltrating cell markers (COR > 0.2) BDCA-1, GATA3, and BCL6 has not been supported in the literature and requires further study. In conclusion, our study shows that ERBBs and M1 Macrophage, Dendritic cell, Th1, Th2, Th17, and Treg cells have a more significant relationship (Cor > 0.2) in cutaneous melanoma. Together, these findings uncover that ERBBs may play an essential role in recruitment and supervision.

MDSCs are the heterogeneous population of immature bone marrow cells derived from bone marrow (Dumitru et al., 2012), which are composed of bone marrow progenitors, immature macrophages, immature granulocytes, and immature dendritic cells. MDSCs play a strong immunosuppressive role (Goh et al., 2013; Ostrand-Rosenberg et al., 2017) through their involvement in infection, inflammation, and cancer and have a significant ability to inhibit the T cell response (Gabrilovich and Nagaraj, 2009; Scapini et al., 2016). In addition to inhibiting the adaptive immune response, MDSCs also regulate the innate immune response by regulating the production of cytokines in macrophages. MDSC also has non-immune functions that promote tumor growth and metastasis by paracrine stimulation of tumor cell proliferation, movement, and angiogenesis (Veglia et al., 2018). Macrophages and myeloid-derived suppressor cells (MDSC), further subdivided into monocytic MDSC (M-MDSC) and polymorphonuclear MDSC (PMN-MDSC) (Cassetta et al., 2019). Human M-MDSC is present in the same density fraction as monocytes but differs from monocytes by the low presence or absence of HLA-DR expression. They are further characterized as lymphocyte lineage marker negative cells with the following phenotype CD11b^+^HLA^–^DR^–^CD14^+^CD15^–^. It is possible to use a CD33 myeloid cell marker instead of a CD11b. Human PMN-MDSC are typically described as CD66b^+^ CD15^+^CD14^–/dim^ CD33^dim^ HLA-DR^–^cells. CD66b or CD15 can be used as lineage markers. PMN-MDSC have been shown to also express other markers, including chemokine markers (e.g., CXCR2, CXCR4), activation markers (e.g., Markers including CD274/PD-L1, CD54/ICAM-1, CD62L, CD63), and functional markers [e.g., arginase 1(ARG1)], at variable levels depending on the disease type and severity (Dumitru et al., 2012; Scapini et al., 2016; Cassetta et al., 2019). MDSCs are associated with a poor prognosis of human melanoma (Meyer et al., 2014). In this study, we found that ERBB2/3 may be closely related to MDSC (Human M-MDSC and Human PMN-MDSC) in cutaneous melanoma. Further experimental studies are needed to support this conclusion, which may be studied by our laboratory in appropriate circumstances, due to the lack of evidence in literature for the direct effects of ERBBs and MDSCs markers in cutaneous melanoma.

In summary, we systematically analyzed ERBBs expression, prognosis, immune infiltration, and its relationship with MDSC. In cutaneous melanoma, ERBB3 high expression and ERBB1/2 low expression were strongly associated with 5 years survival rates of cutaneous melanoma patients but may not affect tumor stage or disease-free survival. ERBB1/2/3 are associated with infiltration of multiple immune cells, especially the M1 Macrophage, Dendritic cell, Th1, Th2, Th17, and Treg cells, which suggests that ERBBs may affect survival rate in cutaneous melanoma patients by affecting immune cell infiltration. Besides, ERBB2/3 are closely related to MDSC, but the role of ERBB2/3 in the cutaneous melanoma population with MDSC remains to be further studied. According to current studies, ERBB1/2/3 may serve as potential therapeutic targets in cutaneous melanoma.

## Data Availability Statement

The original contributions presented in the study are included in the article/Supplementary Material, further inquiries can be directed to the corresponding author/s.

## Author Contributions

YC, RG, and SL conceived and designed this research. SL and RG executed the analysis procedure, contributed analysis tools, and analyzed the results. EL and PZ contributed to the writing of the manuscript. All authors reviewed the manuscript.

## Conflict of Interest

The authors declare that the research was conducted in the absence of any commercial or financial relationships that could be construed as a potential conflict of interest.

## Footnotes

1 https://www.oncomine.org/resource/login.html

2 http://gepia.cancer-pku.cn/index.html

3 https://www.proteinatlas.org/

4 https://www.cbioportal.org/

5 http://metascape.org

6 https://cistrome.shinyapps.io/timer/

7 http://cancer.cellmap.org
