# Supplementary material for: ERBB1/2/3 Expression, Prognosis, and Immune Infiltration in Cutaneous Melanoma
Source: Front Genet. 2021 Mar 1;12:602160. doi: 10.3389/fgene.2021.602160 (PMC7957073; doi:10.3389/fgene.2021.602160)
Supplement: Supplementary file 1 [file Table_1.docx]

**Supplementary Table 1| ERBBs family and 31 genes that neighboring and frequently altered**

| SKCM | | | | | | | | | | | |
| --- | --- | --- | --- | --- | --- | --- | --- | --- | --- | --- | --- |
| Gene markers | ERBB1 | | ERBB2 | | | ERBB3 | | | ERBB4 | | |
|  | Cor | P | Cor | P | | Cor | | P | Cor | | P |
| CBL | 0.1 | * | 0.22 | *** | | 0.059 | | 0.2 | 0.083 | | 0.075 |
| KIT | -0.025 | 0.59 | 0.053 | 0.26 | | 0.076 | | 0.1 | -0.013 | | 0.78 |
| RAF1 | 0.13 | ** | 0.27 | *** | | 0.32 | | *** | 0.076 | | 0.11 |
| PDGFRA | 0.21 | *** | 0.11 | * | | -0.11 | | * | 0.054 | | 0.25 |
| FGFR1 | 0.2 | *** | 0.11 | * | | -0.007 | | 0.88 | 0.14 | | ** |
| SOS1 | 0.036 | 0.44 | 0.14 | ** | | 0.16 | | *** | 0.068 | | 0.14 |
| ALK | -0.017 | 0.72 | -0.0062 | 0.89 | | 0.042 | | 0.37 | -0.019 | | 0.68 |
| IGF1R | -0.053 | 0.25 | 0.11 | * | | 0.19 | | *** | 0.076 | | 0.1 |
| RASA1 | 0.11 | 0.02 | 0.18 | *** | | 0.11 | | * | 0.038 | | 0.42 |
| BRAF | -0.0063 | 0.89 | 0.13 | ** | | 0.26 | | *** | 0.19 | | *** |
| HRAS | 0.45 | 0.34 | 0.13 | ** | | -0.039 | | 0.4 | -0.041 | | 0.38 |
| NRAS | 0.06 | 0.2 | 0.072 | 0.12 | | 0.14 | | ** | 0.05 | | 0.28 |
| ROS1 | 0.38 | *** | 0.12 | ** | | -0.056 | | 0.23 | 0.054 | | 0.24 |
| ARAF | 0.11 | 0.019 | 0.22 | *** | | -0.06 | | 0.2 | 0.02 | | 0.67 |
| KRAS | 0.066 | 0.16 | 0.17 | | *** | 0.11 | * | | 0.032 | 0.49 | |
| NTRK1 | 0.065 | 0.16 | 0.076 | | 0.1 | -0.076 | 0.1 | | -0.012 | 0.8 | |
| RET | 0.055 | 0.24 | 0.091 | | 0.05 | -0.06 | 0.2 | | 0.055 | 0.24 | |
| FGFR3 | 0.029 | 0.54 | 0.15 | | * | 0.065 | 0.16 | | -0.0038 | 0.94 | |
| MAP2K1 | 0.12 | ** | 0.09 | | 0.054 | -0.044 | 0.35 | | 0.088 | 0.059 | |
| RAC1 | 0.027 | 0.57 | 0.059 | | 0.21 | 0.12 | ** | | 0.021 | 0.65 | |
| MAPK1 | -0.018 | 0.71 | 0.23 | | *** | 0.15 | *** | | 0.043 | 0.36 | |
| FGFR4 | 0.051 | 0.28 | 0.0018 | | 0.97 | -0.089 | 0.057 | | -0.019 | 0.69 | |
| PTPN11 | 0.073 | 0.12 | 0.16 | | *** | 0.26 | *** | | 0.089 | 0.057 | |
| NTRK2 | 0.26 | *** | 0.18 | | *** | 0.0027 | 0.95 | | 0.06 | 0.2 | |
| FLT3 | 0.015 | 0.74 | -0.13 | | ** | -0.19 | *** | | -0.021 | 0.66 | |
| FGFR2 | 0.23 | *** | 0.16 | | *** | -0.068 | 0.15 | | -0.0069 | 0.88 | |
| NF1 | 0.0037 | 0.94 | 0.3 | | *** | 0.32 | *** | | 0.16 | *** | |
| ERRFI1 | 0.093 | * | 0.071 | | 0.13 | -0.12 | * | | 0.041 | 0.38 | |
| MAP2K2 | -0.032 | 0.49 | 0.06 | | 0.2 | -0.11 | * | | -0.042 | 0.36 | |
| RIT1 | 0.029 | 0.53 | 0.27 | | *** | 0.37 | *** | | 0.13 | ** | |
| MET | -0.02 | 0.67 | 0.031 | | 0.5 | -0.031 | 0.51 | | 0.023 | 0.62 | |

*: P＜0.05, **: P＜0.01, ***P＜0.001.
